# Marshall H. Webb, D.D.S.

**Published:** 1883-02

**Authors:** 


Marshall Hickman Webb, D. D. S., died at his residence, Lancaster, Pa., on January 1, after a lingering illness, of cancer of the colon, in the 39th year of his age. Dr. Webb was born at Marlsborough, Chester Co., Pa., on October 28, 1844. He studied dentistry with Dr. Frank Hickman, of Coatesville, and graduated from the Philadelphia Dental College in 1867, settling immediately in Lancaster, where he continued in practice up to the time of his last illness. He was a member of the International Medical Congress of 1881, was a valued associate in many different dental societies, and at his death held the position of Lecturer on Operative Dentistry and Dental Histology in the University of Pennsylvania. He left a work on Operative Dentistry (written during his last illness), which will be published posthumously. He was buried, January 4, at West Chester, Pa.

The sad occasion of the death of Dr. Webb demands something more than a mere formal announcement. A singularly quiet, unobtrusive man, his skill as an operator was remarkable. Endowed with unusual inventive genius, he was continually devising new methods and implements, by the use of which complicated operations might be simplified, and with a disinterestedness as rare as it was complete, every product of his ingenious mind was freely given to the profession which he loved and honored. His time was always at the disposal of every brother practitioner, and he would at any time leave a paying patient to gratuitously explain some obscure point in practice to an inquiring dentist. He gave to his brethren all he had—himself; and in the very prime of his days he has laid down his burthen, with life's journey but half completed.

But his untimely death is not all of this sad chapter. In his unselfish zeal, and with the apparent promise of years of usefulness before him, he neglected to make provision for the hour of disaster, and to-day his loved ones—his wife and three children—are exposed to the pitiless storm of adversity. It is no time now to think of what might have been, or to urge his sad fate as a warning and an excuse for illiberality on our part. The profession owes Dr. Webb a debt of gratitude which it is now too late to pay him in person. Let us discharge this honest and just claim by remembering the widow and the fatherless children. If every dentist who has benefited by the unpaid labors of our late associate will but give a tithe of what he has received from him, the family which now knows not where to look for relief will be placed beyond the reach of immediate want. Any of the dental journals will willingly receive and forward contributions, or they may be sent to Dr. J. W. White, at Philadelphia, who will act as treasurer.

---

A London Dentist uses a small incandescent carbon lamp to illuminate the cavity of the mouth during dental operations. It is fitted into a vulcanite cup and covered for safety with a glass shade. The lamp is stated by the inventor, who freely offers its advantages to his fellow dentists, "to give a bright light just where it is needed, without producing undue heat."

